# Investigation of thermally induced damage to surrounding nerve tissue when using curettage and cementation of long bone tumours, modelled in cadaveric porcine femurs

**DOI:** 10.1007/s00402-019-03129-3

**Published:** 2019-02-04

**Authors:** T. A. Murphy, J. A. Mathews, M. R. Whitehouse, R. P. Baker

**Affiliations:** 1 Avon Orthopaedic Centre, Southmead Hospital, North Bristol NHS Trust, Brunel Building, Southmead Road, Bristol, BS10 5NB UK; 2 Musculoskeletal Research Unit, Translational Health Sciences, Bristol Medical School, Southmead Hospital, 1st Floor Learning and Research Building, Bristol, BS10 5NB UK; 3 National Institute for Health Research Bristol Biomedical Research Centre, University Hospitals Bristol NHS Foundation Trust and University of Bristol, Bristol, UK

**Keywords:** Cement, Thermal energy, Long bone tumours, Curettage, Radial nerve damage

## Abstract

**Introduction:**

Curettage with cement augmentation is a technique used in the treatment of bone tumours. Thermal energy released during the cement polymerisation process can damage surrounding tissues. This study aims to record temperature changes at various sites on and around bone during the cementing process. We hypothesised that adjacent structures, such as the radial nerve, may be threatened by this process in the clinical setting.

**Materials and methods:**

Using 18 porcine femurs as a model of the human humerus, we used thermocouples and a thermal imaging camera to measure changes in temperature during the cementing process. Fractures were created in nine samples to establish whether a discontinuity of the cortex had an effect on thermal conduction.

**Results:**

Significantly higher temperatures were recorded in samples with a fracture compared to those without a fracture. The site overlying the centre of the cement bolus (hypothetical site of the radial nerve) demonstrated higher temperatures than all other sites on the same cortex. When considering the radial nerve site, over half the samples demonstrated temperatures exceeding 47 °C for over a minute. When a threshold of 50 °C for more than 30 s was considered, three samples without a fracture exceeded this value compared to two with a fracture.

**Conclusion:**

The temperatures recorded were sufficient to cause damage to neural tissue. Limiting thermal exposure to soft tissues is recommended. Increased attention is required when using larger cement boluses, or where bone quality is poor or a fracture, iatrogenic or preexisting, is present.

## Introduction

Curettage and polymethylmethacrylate (PMMA) bone cement implantation can be used in the surgical management of lytic lesions of long bones [1]. Thermal energy is released during the exothermic polymerisation process of cement curing. Thermal energy has the beneficial effect of killing any remaining tumourous cells and reducing the incidence of recurrence [2–4]; however, increases in temperature can lead to bone necrosis [4, 5], chondrocyte damage [6], and even skin damage [7].

PMMA cement used in vertebroplasty has been linked with damage to surrounding soft tissue, including the adjacent nerve roots [8]. Thermal damage to nerve tissue is dose and time dependent in rat models at temperatures of 43–45 °C [9] and in porcine nerve tissue, temperatures of 60–70 °C for 5 min have demonstrated severe neural degeneration [10].

We hypothesised that treatment of lytic bone lesions at the level of the spiral groove treated with curettage and cementing may expose the radial nerve to elevated temperatures. Cases of radial nerve palsy have been seen when cement is used in surgical procedures involving the humerus, in particular revision arthroplasty [11]. We sought to find the maximum temperatures generated during the cement polymerisation process.

In this study, we used porcine femurs as a model due to their morphological similarity in the diaphysis to the human humerus. 15–20% of patients presenting with a giant cell tumour have a fracture [3]. We further hypothesised that a fracture, or a breach in the cortex, could lead to increased thermal exposure of the overlying nerve that may be sufficient to increase the risk of neurological damage.

## Methods

### Procedure

**Fig. 1:**
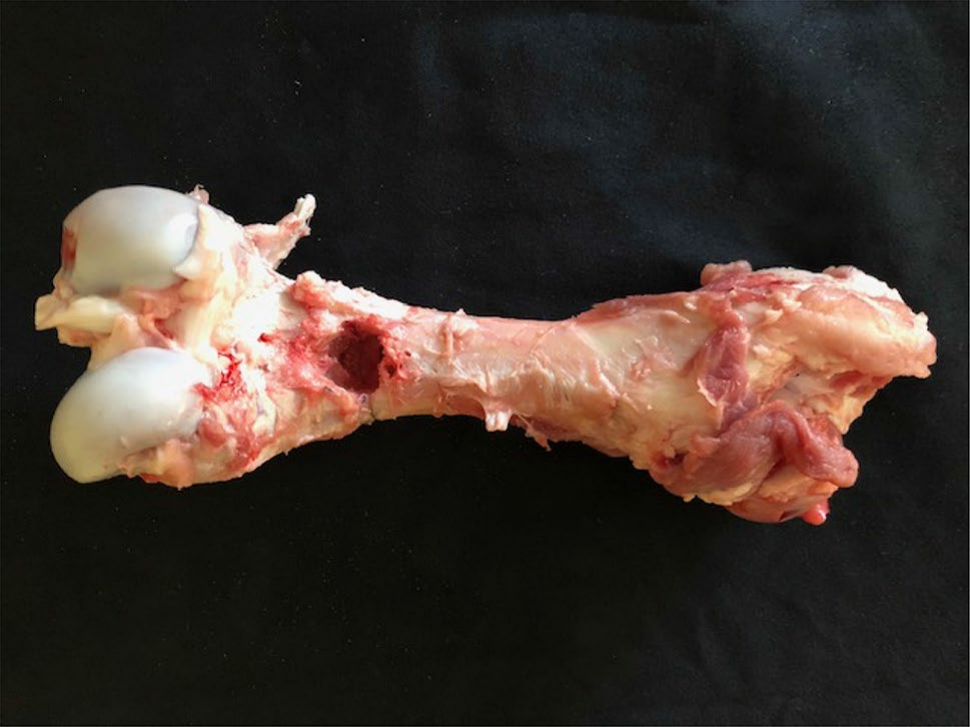
Porcine femur showing 12-mm/12-mm window

**Fig. 2:**
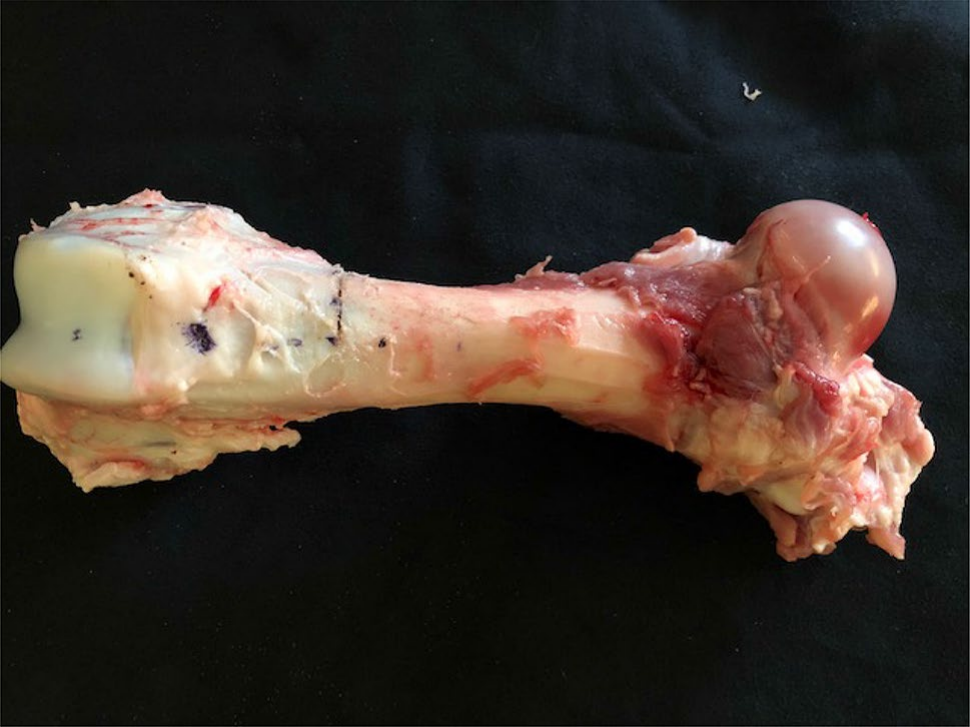
Porcine femur demonstrating ‘fracture line’ extending to anterior cortex

18 porcine femurs were obtained from a local abattoir. To prepare the lytic defects, for each specimen, square windows (12 mm sides) were cut into the dorsal cortex just proximal to the femoral condyles using a 2.5-mm drill and osteotomes (see Fig. 1). Via this window, bone and marrow were curetted out until the cavity could accommodate 15 ml of water (15 cm^3^, with water level flush with the outer cortex). 9 of the 18 bones were prepared to simulate a fracture at the cavity site. The fractures were produced by cutting into the bone using a fine saw blade to produce a discontinuity in the cortex. Fracture lines were of full cortical thickness. To be reproducible, all fracture lines extended from the mid-point of the window on the dorsal aspect of the bone circumferentially to the mid-point of the bone on the anterior cortex perpendicular to the longitudinal axis (Fig. 2).

**Fig. 3:**
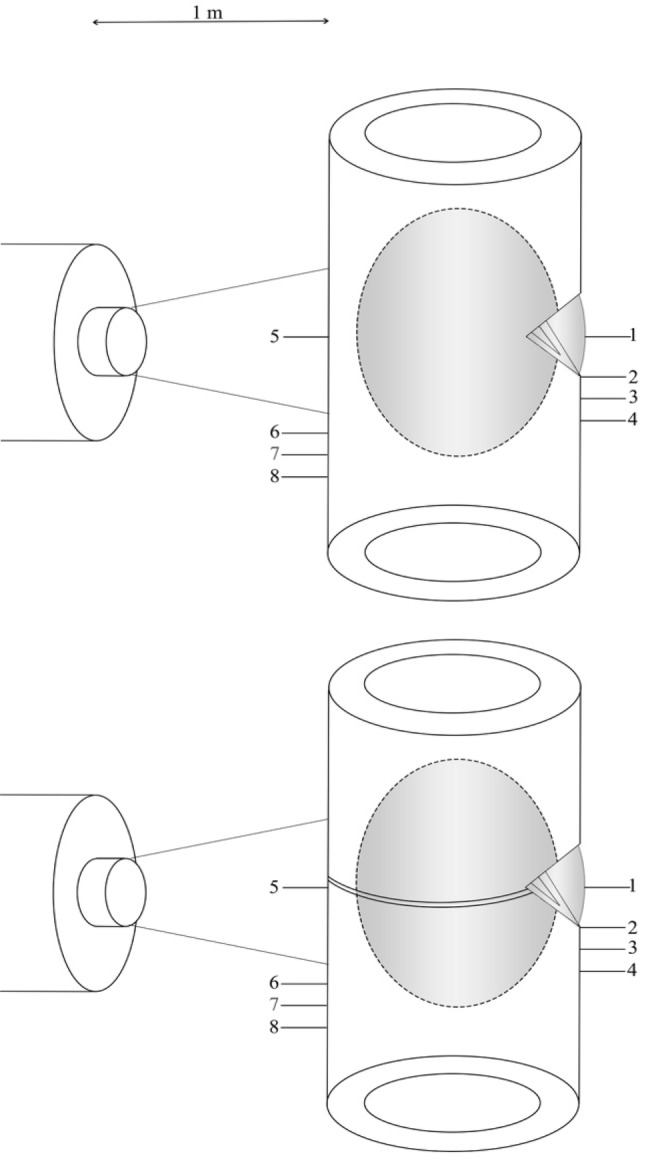
Demonstrates experimental set-up, with and without fracture

The bone was stabilised to the work surface using two bench clamps. A system of eight thermocouples was then used to measure temperatures at the following sites: anterior aspect, cement surface, cement–bone interface, 5 mm from cement–bone interface, 10 mm from cement–bone interface. Thermocouples were then placed on the dorsal cortex (opposite side to the window) at the following sites: mid-point of cement bolus (worst case scenario position of radial nerve), proximal edge of cement bolus, 5 mm from proximal edge of cement bolus, and 10 mm from proximal edge of cement bolus (Fig. 3). Data from the thermocouples were processed by an 8-port Thermocouple Datalogger (PICO technology TC-08; St Neots, England). Data were recorded in an Excel spreadsheet (Microsoft Corporation, Redmond, Washington, USA).

**Fig. 4:**
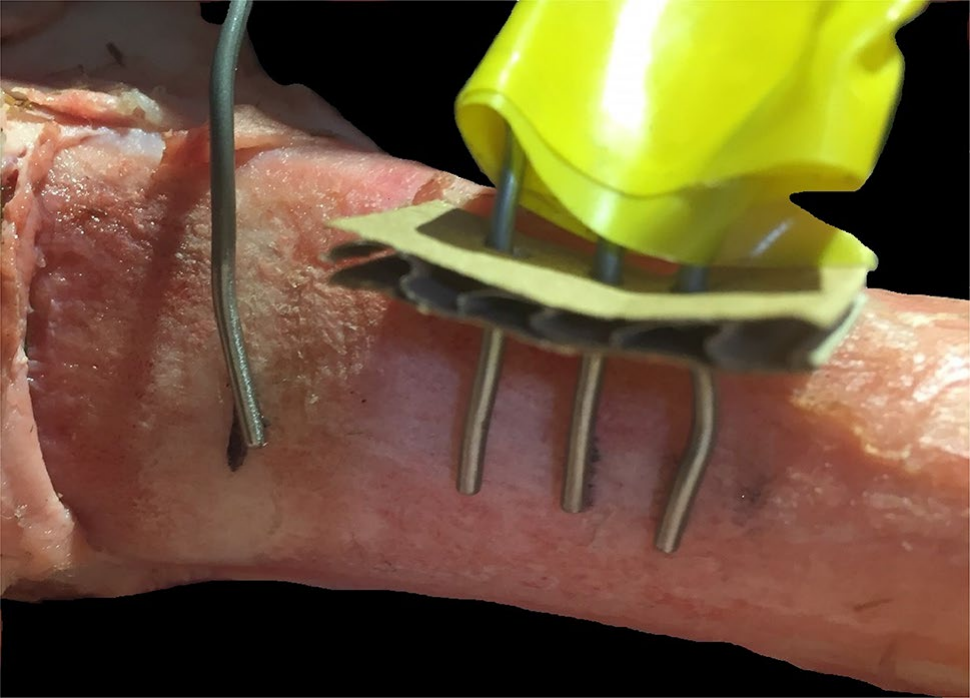
Showing thermocouple set-up at proposed site of radial nerve

A thermal imaging camera (FLIR systems, West Malling, England) was placed 1 m from the anterior cortex and centred over the position of the cement bolus. Images were taken every 20 s. The emissivity value was set to 0.99 [12] (Fig. 4).

**Fig. 5:**
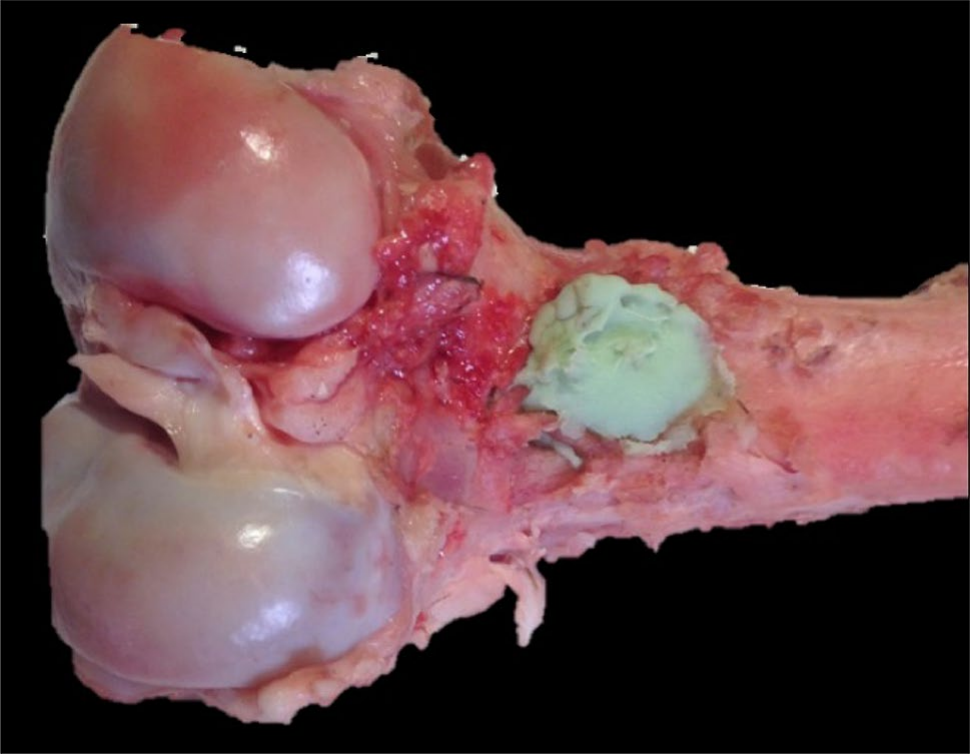
Porcine femur showing cavity filled with cement

Palacos (Heraeus, Wehreim, Germany) bone cement with gentamicin was vacuum mixed following the manufacturer’s instructions. Cement was injected into the cavity and finger pressurisation was used to ensure the cavity was filled and a 15 cm^3^ bolus was achieved. The surface of the bolus was left flush with the cortex as far as possible to leave minimal cement outside the bone. Cement did not extrude from the fracture lines (Fig. 5).

**Fig. 6:**
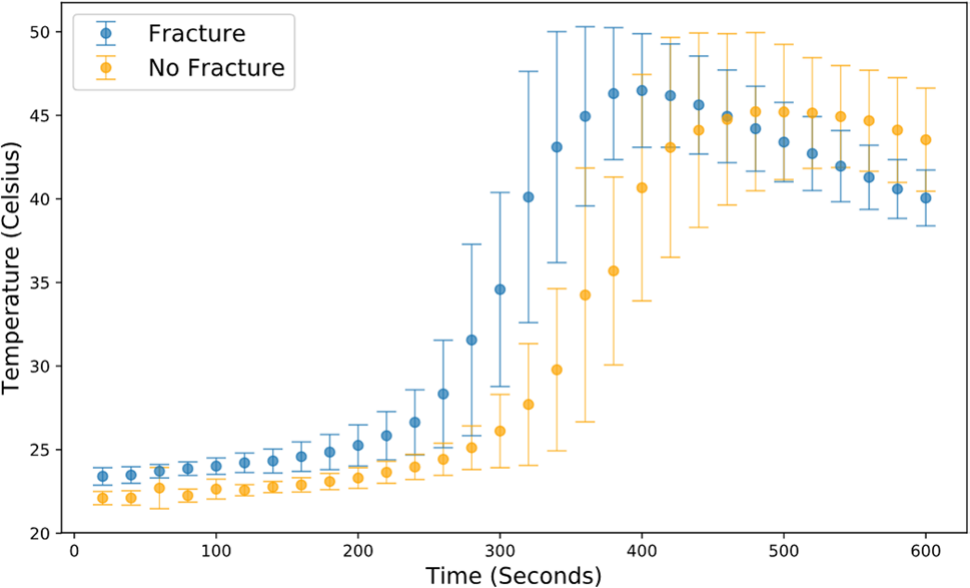
Graph showing thermal imaging camera data against time with 95% confidence intervals plotted. Programme used: statsmodels.org and matplotlib version 3.0.2

**Fig. 7:**
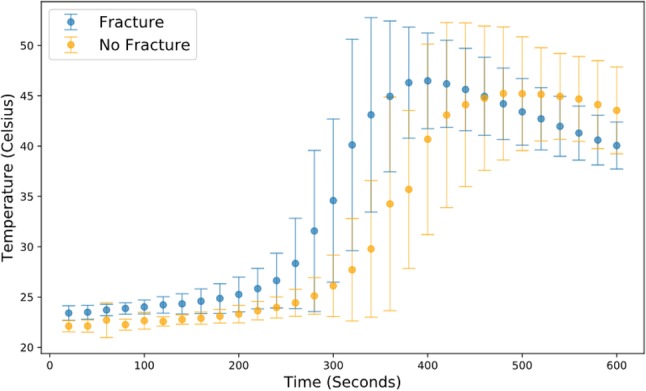
Graph showing thermal imaging camera data against time with one standard deviation plotted. Programme used: pandas.pydata and matplotlib version 3.0.2

Data collection was commenced at 300 s after the initiation of cement mixing and continued for 600 s (10 min). Data were collected at 10-s intervals.

### Statistical methods

A power calculation was performed on data from a previous pilot study performed by our group. (Results presented at the British Orthopaedic Oncology Society meeting, Dartmouth-Hitchcock Medical Centre, Oxford, 2015). In this study, 12 samples were prepared with a curetted lesion and unintentional fractures occurred in 2 of the specimens at the site of the curetted lesion. The mean temperature recorded on the dorsal cortex in the no-fracture specimens was 31.2 °C (standard deviation 2.5) and the mean in the fracture specimens was 42.1 °C giving an effect size of 5.87. For a two-tailed non-parametric test with equal allocation ratio, an alpha value of 0.05 and a power of 0.99 a minimum total sample size of 8 would be required. We decided to use twice this number to insure against data loss during recording or problems with sample preparation.

The D’Agostino and Pearson test was applied to the data to determine whether the data were normally distributed. Correlation between the data recorded by the thermal camera and the thermocouples for each sample was tested with a Spearman rank correlation test. Correlation was ranked as very weak if *r* = 0–0.19, weak if *r* = 0.2–0.39, moderate if *r* = 0.4–0.59, strong if *r* = 0.6–0.79 and very strong if 0.8–1.

Group data were compared with a Mann–Whitney test with two-tailed *p* values. When comparison was made for paired data (e.g. individual thermocouple readings), a Friedman test was used with Dunn’s correction for multiple comparisons.

Categorical data between groups were analysed with a chi-square test and results for relative risk (RR) with 95% confidence intervals (95% CI) are presented.

## Results

For the samples with no fracture, the correlation between the temperatures recorded by the thermal camera and the thermocouples on the near cortex (thermocouples 1–4) was very strong (*r* > 0.8) in all but two thermocouple recordings (*r* = 0.61 and *r* = 0.53). The correlation between the readings for samples with a fracture was strong or very strong (*r* = 0.61–0.99). For thermocouples placed on the far cortex at the site of the radial nerve, the correlation was very strong (*r* > 0.8) in all but one recording (*r* = 0.73), for thermocouples elsewhere on the far cortex, the correlation was less strong and as the distance from the centre of the cement bolus increased, became negative in the majority of cases (Table 1).

**Table 1:** Spearman rank correlations between thermal camera and thermocouple data for each sample (samples 17 and 19 were excluded due to incomplete data)

Sample	Therm 1	Therm 2	Therm 3	Therm 4	Therm 5	Therm 6	Therm 7	Therm 8
1 (no#)	0.94	0.99	0.83	0.61	0.99	0.93	0.61	0.49
2 (no#)	0.98	0.91	0.87	0.85	0.98	0.77	0.46	0.10
3 (no#)	0.95	0.95	0.93	0.89	0.98	0.89	0.86	0.07
4 (no#)	0.99	0.98	0.92	0.92	0.99	0.72	0.47	0.41
5 (no#)	0.95	0.96	0.93	0.93	0.97	0.94	0.89	0.85
6 (no#)	0.87	0.89	0.87	0.87	0.90	0.90	0.90	0.89
7 (no#)	0.98	0.85	0.82	0.53	0.96	0.78	0.69	0.20
15 (no#)	0.96	0.95	0.95	0.94	0.73	0.56	0.25	− 0.58
16 (no#)	0.89	0.93	0.94	0.92	0.97	0.79	0.52	0.13
8 (#)	0.97	0.93	0.90	0.90	0.96	0.65	0.51	− 0.06
9 (#)	0.93	0.91	0.72	0.65	0.91	0.94	0.88	0.78
10 (#)	0.91	0.95	0.76	0.61	0.88	0.82	0.79	0.60
11 (#)	0.96	0.79	0.74	0.73	0.96	0.81	0.75	0.53
12 (#)	0.99	0.75	0.71	0.69	0.91	0.58	− 0.02	− 0.83
13 (#)	0.98	0.97	0.89	0.67	0.99	0.85	0.43	− 0.67
14 (#)	0.90	0.90	0.88	0.84	0.89	0.31	− 0.08	− 0.79
18 (#)	0.92	0.90	0.87	0.87	0.91	0.88	0.80	0.60
20 (#)	0.95	0.93	0.84	0.82	0.91	0.84	0.84	0.83

When the data between the samples with no fracture were compared to the data for samples with a fracture, as recorded by the thermal camera, significantly higher temperatures were recorded in the fracture group (median 35 °C (IQR 24–44) c.f. 26 °C (IQR 23–44), *p* = 0.028; Table 2).

**Table 2:** Mann–Whitney two-tailed *p* value test for difference between samples with and without a fracture by thermal camera or thermocouple

	Median (IQR) temperature (°C) of samples with no fracture	Median (IQR) temperature (°C) of samples with a fracture	*p* value for Mann–Whitney test
Thermal camera	25.9 (22.8–44.4)	34.6 (24.4–43.7)	0.028
Thermocouple 1	26.2 (21.3–44.2)	35.7 (23.2–42.7)	0.24
Thermocouple 2	21.2 (20.2–29.6)	25.0 (21.7–30.1)	0.0005
Thermocouple 3	20.3 (19.6–25.1)	22.7 (21.2–27.8)	< 0.0001
Thermocouple 4	19.3 (19.0–21.4)	22.7 (21.2–27.8)	< 0.0001
Thermocouple 5	21.6 (20.4–30.2)	25.1 (21.4–30.3)	0.0047
Thermocouple 6	20.3 (20.1–23.3)	21.8 (21.2–26.2)	< 0.0001
Thermocouple 7	20.2 (20.1–21.4)	21.3 (21.1–23.7)	< 0.0001
Thermocouple 8	20.0 (19.8–20.4)	21.1 (20.8–21.6)	< 0.0001

When the data for the individual thermocouples were compared between samples with a fracture or no fracture, there was no significant difference for thermocouple 1 (cement surface temperature; *p* = 0.24). There was a statistically significant difference for all other thermocouples with the samples with a fracture showing higher temperatures than samples without a fracture (*p* ≤ 0.005; Table 2).

When the data from the thermocouples on the far cortex, which are representative of the potential thermal exposure of a nerve overlying said cortex, were considered for samples with no fracture, a significant difference was observed between the thermocouples according to their distance from the centre of the bolus (Friedman test, *p* < 0.0001). The temperature recorded by the thermocouple overlying the centre of the bolus was significantly higher than all of the other thermocouples on the far cortex (Dunn’s multiple comparison test; *p* ≤ 0.0001). When the same comparison was performed for samples with a fracture, the same pattern was repeated (Dunn’s multiple comparison test; *p* ≤ 0.003).

Given the small absolute differences in the observed temperatures, attention was then turned to the time spent in excess of temperatures that may result in nerve damage. Due to strong correlation between the two measurement modalities, we have opted to use only the thermal camera data to present this. For the data recorded by the thermal camera, five out of nine samples without a fracture with complete data recorded a temperature in excess of 47 °C for more than 1 min compared to four out of nine samples with a fracture [*p* = 1.00; RR = 1.25 (0.49–3.12)]. When a threshold of 50 °C for more than 30 s was considered, three samples without a fracture exceeded this value compared to two with a fracture [*p* = 1.00; RR = 1.50 (0.32–6.95)] (see Fig. 6 in Appendix 1 and Fig. 7 in Appendix 2). No samples in either group exceeded a threshold of 55 °C. When the far cortex thermocouple data were considered (thermocouples 5–8), no samples exceeded 47 °C.

## Discussion

The results demonstrate the elevated temperatures that surrounding tissues are subjected to during the cementing process. Temperatures achieved at the site of the overlying radial nerve were well above body temperature. In five out of nine samples without a fracture and four out of nine samples with a fracture the thermal camera recorded temperatures exceeding 47 °C for over 1 min. The maximum temperatures demonstrated by thermocouple 5, the hypothetical position of the radial nerve, were reassuring overall.

The samples with fractures demonstrated significantly higher average temperatures in all thermocouples and the thermal camera data, suggesting that the fractures lead to increased conduction of thermal energy, but interestingly the no-fracture group was shown to maintain thermal energy for longer. We speculate that discontinuity in the cortex leads to faster dissipation of thermal energy.

Multiple studies have demonstrated tissue damage to bone and cartilage as a result of thermal energy from bone cement [2–4]. Whilst neural tissue damage has been postulated in the use of cement in vertebroplasty [8], the possible effects of cementation on local peripheral nerves is unclear.

Damage to nerve tissue has been demonstrated at temperature of 43–45 °C [9]; however, this was shown to be time dependent. The temperatures reached in both the fracture and no-fracture group were reassuring relative to this regarding thermocouple 5. The thermal camera did demonstrate higher temperatures exceeding 47 °C. However, despite this we do not believe this would lead to an increased risk of radial nerve damage due to the limited time over which the radial nerve would be exposed to excessive temperatures. In addition, thermal camera data depicting maximum temperature would only have been representative of a small area of bone and would likely not have affected a significant proportion of the traversing radial nerve. A discontinuity on the cortex of the bone may subject the nerve to further rises in temperature. But again we would suggest that the risk of damage to the nerve, whilst possible, would be low seeing as the fractured bones appeared to dissipate thermal energy more quickly than the non-fractured bones.

There are limitations of this study that must be considered when interpreting the results. The bones used were from differing porcine subjects and, therefore, there may have been a variation in general dimensions and cortical thickness. This made creating completely equal fracture sizes difficult, whilst we were certain of cement bolus size, controlling the distribution of the cement bolus was more challenging. The study could be repeated with imaging of each subject to assess the distribution of each cement bolus. The cement bolus size used was small at 15 cm^3^. In theory, larger cement boluses may be used in the clinical setting. Larger boluses are likely to produce more thermal energy. In addition, we have no data on the quality of the bone used or histological analysis of the specimens after experimentation. Whilst we considered the porcine femur to be a close representation of the human humerus in size, we have to bear in mind that it is a weight-bearing bone and may have very different qualities in terms of cortical thickness and bone mineral density. Conversely, whilst efforts were made to limit heterogeneity between test subjects during this project, patients presenting with tumours of the humerus will have a broad range of characteristics.

Whilst the results of this experimental study are reassuring with regard to the possibility of radial nerve damage due to thermal injury during curettage and cementing of lytic lesions of the humerus, we would advise that caution is taken when large boluses are used or the nerve directly overlies the site.

In cases with added variables, for example, larger cement boluses, osteoporotic bone with thin cortices, increased bone loss or extensive fracture patterns; the potential of risk of thermal injury to adjacent structures to the bone should be considered.

## Appendix 1

See Fig. 6.

## Appendix 2

See Fig. 7.

## References

[1] Agarwal MG, Nayak P (2015). Management of skeletal metastases: an orthopaedic surgeon’s guide. Indian J Orthop.

[2] Mermerkaya MU, Bekmez S, Karaaslan F (2014). Intralesional curettage and cementation for low-grade chondrosarcoma of long bones: retrospective study and literature review. World J Surg Oncol.

[3] van der Heijden L, Dijkstra PD, van de Sande MA (2014). The clinical approach toward giant cell tumor of bone. Oncologist.

[4] Nelson DA, Barker ME, Hamlin BH (1997). Thermal effects of acrylic cementation at bone tumour sites. Int J Hyperthermia.

[5] Mjöberg B, Pettersson H, Rosenqvist R, Rydholm A (1984). Bone cement, thermal injury and the radiolucent zone. Acta Orthop Scand.

[6] Radev BR, Kase JA, Askew MJ, Weiner SD (2009). Potential for thermal damage to articular cartilage by PMMA reconstruction of a bone cavity following tumor excision: a finite element study. J Biomech.

[7] Arumilli BR, Paul AS (2007). Pretibial full thickness skin burn following indirect contact from bone-cement use in a giant cell tumour. Sarcoma.

[8] Lai PL, Tai CL, Chen LH, Nien NY (2011). Cement leakage causes potential thermal injury in vertebroplasty. BMC Musculoskelet Disord.

[9] De Vrind HH, Wondergem J, Haveman J (1992). Hyperthermia-induced damage to rat sciatic nerve assessed in vivo with functional methods and with electrophysiology. J Neurosci Methods.

[10] Konno S, Olmarker K, Byröd G (1994). Acute thermal nerve root injury. Eur Spine J.

[11] Lee JS, Kim JY, Jung HJ, Jung HS, Baek JH (2013). Radial nerve recovery after thermal injury due to extruded cement during humeral revision in total elbow arthroplasty. J Shoulder Elbow Surg.

[12] Baker R, Whitehouse M, Kilshaw M (2011). Maximum temperatures of 89°C recorded during the mechanical preparation of 35 femoral heads for resurfacing. Acta Orthop.

